# Soft Tissue Sarcoma: The Predominant Primary Malignancy in the Retroperitoneum

**DOI:** 10.1080/13577140120048881

**Published:** 2001-03

**Authors:** Thijs van Dalen, Jan-Willem W. Coebergh, Mariel K. Casparie, Charles H. F. Gimbrère, Harald J. Hoekstra, Bert N. Van Geel, Frits Van Coevorden, Adriaan Hennipman

**Affiliations:** 1 Dutch Network and National Database for Pathology (PALGA) Utrecht The Netherlands; 2 Netherlands Cancer Registry Vereniging van Integrale Kankercentra Utrecht The Netherlands; 3 Dutch Soft Tissue Sarcoma Group Vereniging van Integrale Kanker Centra Utrecht The Netherlands; 4 Department of Surgery Universitair Medisch Centrum Room G04.228 Heidelberglaan 100 Utrecht 3584 CX The Netherlands

## Abstract

*Purpose.* In the clinical work-up of a retroperitoneal mass, the diagnosis of soft tissue sarcoma is often not considered. Incidence rates of various malignant and benign retroperitoneal tumours were studied to determine the incidence of soft tissue sarcoma in comparison with other neoplasms in the retroperitoneal space.

*Method.* Nation-wide data on retroperitoneal tumours, collected prospectively over a 5-year period (1 January 1989– 1 January 1994), were supplied by the Netherlands Cancer Registry and The Dutch Network and National Database for Pathology.

*Results.* Seven hundred and six patients with a primary retroperitoneal neoplasm were identified; 566 patients had a malignant tumour (80%). A soft tissue sarcoma (STS) was the most frequently diagnosed malignant tumour (*n* = 192), The agestandardised incidence of retroperitoneal STS was 2.5 per million person-years. The male/female ratio for STS was 0.73. In females, STS comprised 41%of all malignant retroperitoneal tumours, carcinoma of unknown primary tumour site (CUP) comprised 31%, and malignant lymphomas (ML) comprised 22%, whereas in males these values were 28% (STS), 30% (CUP), and 32% (ML), respectively.

*Discussion.* Soft tissue sarcomas, albeit rare, are relatively common primary tumours in the retroperitoneum, especially in women.


*Sarcoma* (2001) **5**, 5–8

ORIGINAL ARTICLE

### Soft tissue sarcoma: the predominant primary malignancy in the retroperitoneum

THIJS VAN DALEN,* JAN-WILLEM W. COEBERGH,* MARIEL K. CASPARIE,[1] CHARLES H. F. GIMBR¨ RE,[2] HARALD J. HOEKSTRA,* BERT N. VAN GEEL,* FRITS VAN COEVORDEN* & ADRIAAN HENNIPMAN,* FOR THE DUTCH SOFT-TISSUE SARCOMA GROUP

*[1]Dutch Network and National Database for Pathology (PALGA), Utrecht, The Netherlands, [2]Netherlands Cancer Registry, Vereniging van Integrale Kankercentra, Utrecht, The Netherlands, and *Members of the Dutch Soft Tissue Sarcoma Group, Vereniging van Integrale Kanker Centra, Utrecht, The Netherlands*

**Abstract**

*Purpose.* In the clinical work-up of a retroperitoneal mass, the diagnosis of soft tissue sarcoma is often not considered. Incidence rates of various malignant and benign retroperitoneal tumours were studied to determine the incidence of soft tissue sarcoma in comparison with other neoplasms in the retroperitoneal space.

*Method.* Nation-wide data on retroperitoneal tumours, collected prospectively over a 5-year period (1 January 1989–1 January 1994), were supplied by the Netherlands Cancer Registry and The Dutch Network and National Database for Pathology.

*Results.* Seven hundred and six patients with a primary retroperitoneal neoplasm were identified; 566 patients had a malignant tumour (80%). A soft tissue sarcoma (STS) was the most frequently diagnosed malignant tumour ($n = 192$), The age-standardised incidence of retroperitoneal STS was 2.5 per million person-years. The male/female ratio for STS was 0.73. In females, STS comprised 41% of all malignant retroperitoneal tumours, carcinoma of unknown primary tumour site (CUP) comprised 31%, and malignant lymphomas (ML) comprised 22%, whereas in males these values were 28% (STS), 30% (CUP), and 32% (ML), respectively.

*Discussion.* Soft tissue sarcomas, albeit rare, are relatively common primary tumours in the retroperitoneum, especially in women.

**Key words**: *incidence, primary cancer, retroperitoneum, soft tissue sarcoma*

### Introduction

Primary tumours in the retroperitoneal space that do not originate from the retroperitoneal viscera are uncommon. Soft tissue sarcomas (STS) arise occasionally in the retroperitoneum, but are so rare that the diagnosis is not always taken into account by a clinician who is investigating a patient with a retroperitoneal neoplasm. In a population-based study on the clinical presentation of patients with a retroperitoneal STS, more than one-third of the patients was initially diagnosed erroneously, and operated for a tumour that was not considered to be a STS.[1]

Data on the frequency of STS and other primary malignancies in the retroperitoneal space are scarce,[2–4] and recent population-based figures are not available. We therefore estimated incidence rates of STS in relation to other primary non-visceral tumours in the retroperitoneum with the help of two national registries.

### Methods

Data on patients in The Netherlands with retroperitoneal neoplasms that were newly diagnosed and confirmed histologically between 1 January 1989 and 1 January 1994 were retrieved from two sources.

The Netherlands Cancer Registry (NCR) manages a databank with information on all patients with newly diagnosed cancers. Malignancies are coded according to the International Classification of Diseases for Oncology.[5] Data were provided on patients identified within the NCR database as having retroperitoneally localised STS, seminoma (non-testicular), teratoma, and primary (epidermoid) carcinoma, and were used to calculate their respective incidence.

Because of the aforementioned classification used by the NCR,[5] some malignant neoplasms that can occur in the retroperitoneum are not topographically identifiable as such (e.g. retroperitoneal lymphoma is coded topographically as an abdominal tumour). Therefore, additional information was provided by

Correspondence to: Th. van Dalen, Department of Surgery, Universitair Medisch Centrum, Room G04.228, Heidelberglaan 100, 3584 CX Utrecht, The Netherlands. E-mail: nounou@knoware.nl

1357–714X print/1369–1643 online/01/010005–04 © 2001 Taylor & Francis Ltd
DOI: 10.1080/13577140120048881

The Dutch Network and National Database for Pathology (PALGA), which contains standardised abstracts of all pathology reports in The Netherlands, with computerised data submission by the individual pathology laboratories. The latter registry classifies pathological conditions according to the Systematized Nomenclature of Medicine (SNOMED, American College of Pathologists), and allows a search for all malignant and benign neoplasms in the retroperitoneal space.

Crude incidence rates and age-specific incidence rates for 10-year age groups were calculated per million person-years for males and females using the Dutch population on 1 July 1991 (7,478,911 males and 7,648,088 females; source, Statistics Netherlands).

### Results

Seven hundred and six patients were identified as having a primary non-visceral retroperitoneal tumour (Table 1). The majority of these tumours was of malignant origin ($n$ = 566; 80%). STS was the most common non-visceral malignant tumour in the retroperitoneum ($n$ = 192; 34% of the malignant tumours). The crude incidence of retroperitoneal STS was 2.5 per million person-years, and the male to female ratio was 0.73. STS was the most common malignant tumour in females (41%), followed by carcinoma of unknown primary tumour site (CUP) (31%), and malignant lymphoma (ML) (22%). In males, these proportions were 28 (STS), 30 (CUP), and 32% (MI), respectively.

In Figure 1, the age-specific incidence of primary

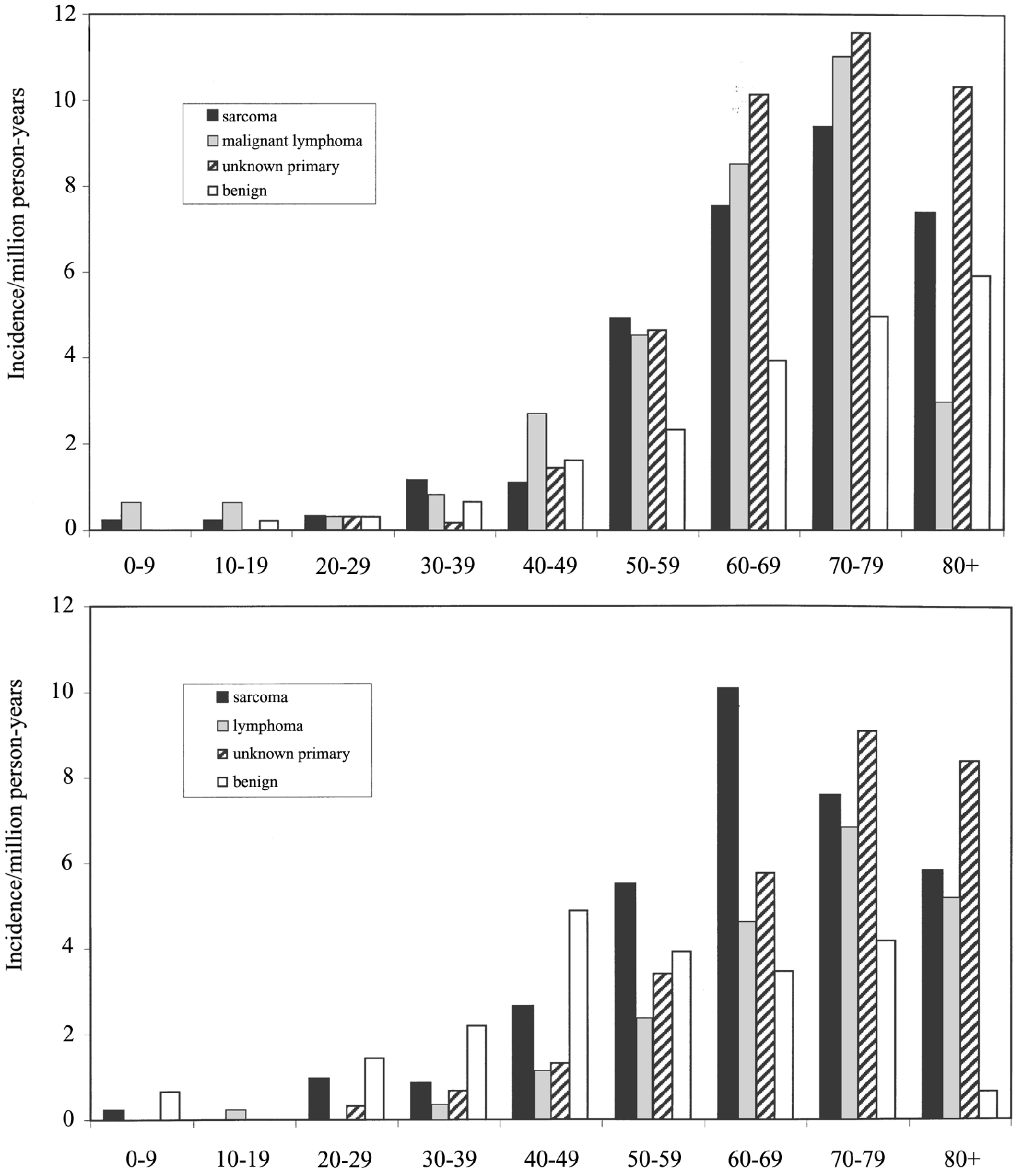

**Fig. 1.** *Primary (non-visceral) retroperitoneal tumours in (a) males and (b) females: age-specific incidence per million person-years in The Netherlands (1989–1993).*

non-visceral retroperitoneal tumours is delineated for males and females. For convenience, benign retroperitoneal tumours were grouped together. The age-specific incidence showed a similar pattern in both sexes for all three common malignant tumours: an increase in middle age followed by a levelling thereafter. In females, STS was more common than ML irrespective of age, and only less frequent than CUP in elderly women. CUP and ML more often occurred in men than in women and, particularly in males aged between 60 and 80 years, both CUP and ML more commonly occurred than STS.

### Discussion

In this population-based study, 80% of all primary non-visceral tumours in the retroperitoneum were malignant. Soft tissue sarcomas comprised one-third of the malignant tumours and were predominant in females. Malignant lymphoma and carcinoma of unknown primary tumour site made up most of the remainder. An age-related incidence rise was seen for all three malignancy types in both sexes.

The crude incidence of 2.5 per million person-years resulted in 40 new patients with retroperitoneal STS annually in the Netherlands. In our country, approximately 700 surgeons are affiliated to 110 hospitals. This would imply that one patient with a retroperitoneal STS is seen only once every 3 years in the average surgical practice, assuming that all patients with retroperitoneal STS are referred to surgeons. In reality, they are not all seen by surgeons because some will be managed by other specialists (e.g. urologists or gynaecologists).

This unfamiliarity with STS in the retroperitoneum is likely to contribute to the difficulties in establishing the diagnosis correctly. In the aforementioned population-based study,[1] more than one-third of the patients with a retroperitoneal STS was operated for assumed other pathological conditions; STS were rarely if ever confused with malignant lymphomas or carcinomas of unknown primary tumour site. They were rather mixed up with renal carcinomas and tumours of the female reproductive organs. The much higher incidence of the latter tumours (Table 2) is a probable explanation for this.

Yet, for the clinician who is examining a patient with a retroperitoneal tumour, radiological techniques such as computed tomography or magnetic resonance imaging/magnetic resonance angiography should readily discriminate visceral from non-visceral neoplasms.[6–8] Subsequently, pathological examination should be of help to discriminate between STS, malignant lymphoma, and carcinoma of unknown primary site. Fine needle aspirates and core needle biopsies may confirm the presence of a STS,[9–11] but the yield in the case of STS is limited.[10,12] More importantly, needle biopsies can reliably distinguish a carcinoma of unknown primary tumour site,[13] and the lymphoid nature of a retroperitoneal tumour.[14,15] Hence, the main value of a biopsy is to exclude the latter two malignancies and, in that respect, the value of an open surgical biopsy becomes questionable.

In conclusion, at the unusual occasion that a clinician is confronted with a non-visceral neoplasm in the retroperitoneal space, the tumour is most probably malignant, and a soft tissue sarcoma is relatively

**Table 1**. *Crude incidence rates of non-visceral retroperitoneal tumours per million person-years in The Netherlands (1989–1993)*

| | Total ($n$) | incidence (per $10^6$ person-years) | Male to female ratio |
|---|---|---|---|
| *Malignant tumours (n = 556)* | | | |
| Soft tissue sarcoma | 192 | 2.5 | 0.73 |
| Carcinoma of unknown primary tumour site | 172 | 2.3 | 1.04 |
| Malignant lymphoma | 154 | 2.0 | 1.52 |
| Seminoma (non-testicular) | 9 | 0.2* | – |
| Malignant teratoma | 13 | 0.2 | 1.6 |
| Primary (epidermoid) carcinoma | 8 | 0.1 | 7 |
| 'Other' | 18 | – | – |
| *Benign tumours (n = 140)* | | | |
| Lipoma | 28 | 0.4 | 0.87 |
| Schwannoma | 24 | 0.3 | 0.25 |
| Leiomyoma | 21 | 0.3 | 0.05 |
| Paraganglioma | 12 | 0.2 | 3 |
| Neurofibroma | 8 | 0.1 | 0.33 |
| Lymfangioma | 8 | 0.1 | 1.67 |
| Haemangioma | 8 | 0.1 | 1 |
| Ganglioneuroma | 7 | 0.1 | 0.4 |
| Neurilemmoma | 6 | 0.1 | 0.5 |
| Phaeochromocytoma (non-adrenal) | 6 | 0.1 | 5 |
| Other | 12 | – | 0.38 |

* Incidence per million person-years in men.

**Table 2.** *Crude incidence rates of malignant tumours of the retroperitoneal and female reproductive organs per million person-years in The Netherlands (1989–1993)*[16,17]

| | Total (*n*) | Incidence (per $10^6$ person-years) | Male to female ratio |
|---|---|---|---|
| Renal carcinoma | 6665 | 88.1 | 1.46 |
| Adrenal carcinoma | 231 | 3.1 | 0.94 |
| Ureteric/pelvic carcinoma | 1256 | 16.6 | 1.84 |
| Pancreatic carcinoma | 6772 | 89.5 | 1.04 |
| Cancer of the ovary | 6173 | 161.* | – |
| Cancer of the corpus uteri | 6438 | 168.* | – |
| Retroperitoneal soft tissue sarcoma | 192 | 2.5 | 0.73 |

* Incidence per million person-years in women.

common in comparison with other primary malignancies. When a lymphoma or an unknown primary carcinoma can be ruled out, a retroperitoneal mass most likely is a soft tissue sarcoma. The clinical implication of this finding is important. Contrary to the medical treatment of lymphomas and unknown primary carcinomas, surgery is the main therapeutic modality in the treatment of retroperitoneal soft tissue sarcoma. As a consequence, a retroperitoneal mass of unknown nature deserves a secure perioperative strategy assuming the presence of a STS.

### References

1 van Dalen TH, van Geel AN, van Coevorden F, *et al.* Soft tissue sarcoma in the retroperitoneum: an often neglected diagnosis. *Eur J Surg Oncol* (in press).

2 Pinson CW, ReMine SG, Fletcher WS, Braasch JW. Long-term results with primary retroperitoneal tumors. *Arch Surg* 1989; 124:1168–73.

3 Johnson AH, Searls HH, Grimes OF. Primary retroperitoneal tumors. *Am J Surg* 1954; 88:155–61.

4 Melicow MM. Primary tumors of the retroperitoneum. A clinico-pathological analysis of 162 cases, review of the literature and tables of classification. *J Int Coll Surg* 1953; 19:401–49.

5 *International Classification of Diseases for Oncology*, 2nd edn. Geneva: World Health Organisation, 1990.

6 Storm FK, Mahvi DM. Diagnosis and management of retroperitoneal soft-tissue sarcoma. *Ann Surg* 1990; 214:2–10.

7 Granstrom P, Unger E. MR imaging of the retroperitoneum. *Magn Reson Imaging Clin N Am* 1995; 3:121–42.

8 Meyers MA. *Dynamic Radiology of the Abdomen*, 4th edn. New York: Springer-Verlag, 1994.

9 Costa MJ, Campman SC, Davis RL, Howell LP. Fine-needle aspiration cytology of sarcoma: retrospective review of diagnostic utility and specificity. *Diagn Cytopathol* 1996; 15:23–32.

10 Abdul-Karim FW, Rader AE. Fine needle aspiration of soft-tissue lesions. *Clin Lab Med* 1998; 18:507–40.

11 Ball ABS, Fisher C, Pittam M, Watkins RM, Westbury G. Diagnosis of soft tissue tumours by Tru-Cut biopsy. *Br J Surg* 1990; 77:756–8.

12 Mankin HJ, Mankin CJ, Simon MA. The hazards of the biopsy, revisited. *J Bone Joint Surg Am* 1996; 78:656–63.

13 Mackay BM, Ordonez NG. Pathological evaluation of neoplasms with unknown primary tumor site. *Semin Oncol* 1993; 20:206–28.

14 Wakely PE. Fine needle aspiration cytopathology of malignant lymphoma. *Clin Lab Med* 1998; 18:541–59.

15 Cafferty LL, Katz RL, Ordonez NG, Carrasco CH, Cabanillas FR. Fine needle aspiration diagnosis of intraabdominal and retroperitoneal lymphonas by a morphologic and immunocytochemical approach. *Cancer* 1990; 65:72–7.

16 Visser O, Coeburgh JWW, Schouten LJ, van Dijck JAAM, eds. *Incidence of Cancer in The Netherlands 1996*. Utrecht: Vereniging van Integrale Kankercentra, 2000.

17 *Gynaecological Tumours in The Netherlands 1989–1993*. Utrecht: Vereniging van Integrale Kankercentra, 1997.
